# Reviewer acknowledgement 2013

**DOI:** 10.1186/1471-2393-14-18

**Published:** 2014-01-30

**Authors:** Peter O'Donovan

**Affiliations:** 1 BioMed Central, 236 Gray’s Inn Road, London WC1X 8HB, UK

## Abstract

**Contributing Reviewers:**

The editors of BMC Pregnancy and Childbirth would like to thank all our reviewers who have contributed to the journal in Volume 13 (2013).

## 

Sileshi Abeya

Ethiopia

Peter Abrahams

UK

Babatunde Adedokun

Nigeria

Stuart Adler

USA

Mukesh Mansha Agarwal

United Arab Emirates

Neelam Aggarwal

India

Azar Aghamohamadi

Iran

A. J. Agopian

USA

Yenita Agus

Indonesia

Alexander Aiken

UK

Ashraful (Neeloy) Alam

Australia

Nazmul Alam

Canada

Charles Algert

Australia

Abdel Aziem Ali

Sudan

Tazeen Saeed Ali

Pakistan

Gambo Aliyu

USA

Jill Allison

Canada

Patji Alnæs-Katjavivi

Norway

Joel Ojo Aluko

Nigeria

Charles Ameh

UK

Amanda Ampt

Australia

Annie Anderson

UK

Cindy Anderson

USA

Ngaire Anderson

New Zealand

Stuart Anderson

UK

Alessandro Antonelli

Italy

Iqbal Anwar

Bangladesh

Edward Araujo Júnior

Brazil

Megan Arbour

USA

Madelynne Arden

UK

Ana Areia

Portugal

Victoria Arija

Spain

Catherine Arnaud

France

Jose-Antonio Arraztoa

Chile

Nega Assefa

Ethiopia

Neil Avent

UK

Pinar Ay

Turkey

Stella Babalola

USA

Peter Baghurst

Australia

Marie-Clare Balaam

UK

Anita Banerjee

UK

Faraj Barah

Portugal

Flora Maria Barbosa Da Silva

Brazil

Lesley Barclay

Australia

Janine Barden-O'Fallon

USA

Pierre Barker

USA

Suzanne Barr

UK

Jose Luis Bartha

Spain

Linda Bartlett

USA

Robert Basaza

Uganda

Jamie Bastek

USA

Pat Bazeley

Australia

Franziska Becker

Germany

Mulu Muleta Bedane

Ethiopia

Katrien Beeckman

Belgium

Roxana Behruzi

Canada

Yaakov Beilin

USA

Francesco Bellanti

Italy

David Bellinger

USA

Jogchum Beltman

Netherlands

Gervais Beninguisse

Cameroon

V Berghella

USA

João Bernardes

Portugal

Annette Bernloehr

Germany

Peter Berti

Canada

Ana Pilar Betran

Switzerland

Maria Enrica Bettinelli

Italy

Solomon Beza

Ethiopia

Sibhatu Biadgilign

Ethiopia

Debra Bick

UK

C. Lesley Biggs

Canada

Zewdie Birhanu

Ethiopia

Mary Anne Biro

Australia

Elisabeth Krefting Bjelland

Norway

Mairead Black

UK

Vivian Black

South Africa

Andrew Blann

UK

Michael Bloch

USA

Eric Blyth

UK

Agatha Boerleider

Netherlands

Francisco Bolumar

Spain

Dolly Bondarianzadeh

Iran

Amy Bonomi

USA

Matthias Borchert

Germany

Angela Bowen

Canada

Coleen Boyle

USA

Kristin Brække

Norway

Gerard Breart

France

Katie Bristow

UK

Tim Bruckner

USA

Traolach Brugha

UK

Deborah Bruns

USA

Louise Bryant

UK

Helen Bryers

UK

Sherri Bucher

USA

Eckhart Buchmann

South Africa

Pradeep Buddharaju

India

Jorge Burgos

Spain

Melanie Bussey

New Zealand

Bridgette Byrne

Ireland

Luis Cabero

Spain

David Cahill

UK

John Alexander Cairns

UK

Leonie Callaway

Australia

Mary Cameron

USA

Michael Cannon

USA

Joseph Canterino

USA

Yunxia Cao

China

Giampiero Capobianco

Italy

Angela Carberry

Australia

Mary Carolan-Olah

Australia

Gaspare Carta

Italy

Ben Carter

UK

Jose G Cecatti

Brazil

Tammy Chang

USA

Andrew Amos Channon

UK

Lama Charafeddine

Lebanon

Picklu Chaudhuri

India

Helen Cheyne

UK

Pragti Chhabra

India

Shakuntala Chhabra

India

Joanne Chiwaula

Ghana

Thanapan Choobun

Thailand

Wendy Christiaens

Belgium

Parul Christian

USA

Olivier Claris

France

Elizabeth Cluett

UK

Teresa Cobo

Spain

Judy Slome Cohain

Israel

Christine Connors

Australia

Peter Cooper

South Africa

Michael Coory

Australia

Maria Laura Costa

Brazil

Anthony Costello

UK

Barbara Coughlan

Ireland

Kim Cox

USA

Suzanne Cox

USA

David Cromwell

UK

Sarah Crozier

UK

Andrew E. Czeizel

Hungary

Hannah Dahlen

Australia

Amanda Daley

UK

Anne Kjersti Daltveit

Norway

Deirdre Daly

Ireland

Stefania D'Angelo

UK

Ina Danquah

Germany

Maureen Dar Iang

Nepal

Shrinivas Darak

India

Kaberi Dasgupta

Canada

Mary-Ann Davey

Australia

Deborah Davis

Australia

Manuela De Allegri

Germany

Irene De Graaf

Netherlands

Ank De Jonge

Netherlands

Jan Willem De Leeuw

Netherlands

Catalina De Paco

Spain

Claire De Vienne

France

Toity Deave

UK

Eugene Declercq

USA

Thérèse Delvaux

Benin

Anna Dencker

Sweden

Kebede Deribe

Ethiopia

Jean-Claude Desenclos

France

Andrew Dewan

USA

Elaine Dietsch

Australia

Berna Dilbaz

Turkey

Umut Dilek

Turkey

Angel Dillip

Tanzania

Mai Do

USA

Jodie Dodd

Australia

Maman Dogba

Canada

Serena Donati

Italy

Donna Dowling

USA

Soo Downe

UK

Therese Dowswell

UK

Evemie Dubé

Canada

Genevieve Dumas

Canada

Kirsty Dundas

UK

Dat Duong

Viet Nam

Corinne Dupont

France

Jill Durocher

USA

Johannes Duvekot

Netherlands

Alexandra Duxbury

UK

Fiona Dykes

UK

Kathy Eagar

Australia

Christine East

Australia

John Eastwood

Australia

Karen Eden

USA

Leroy Edozien

UK

Rodney Edwards

USA

Maria Ekelin

Sweden

Alison El Ayadi

USA

Offer Erez

Israel

Carola Eriksson

Sweden

Jimmy Espinoza

USA

Kelly Evenson

USA

Thomas Everett

UK

Christine Faerber

Germany

Alexandre Faisal-Cury

Brazil

Sara Farchi

Italy

Cynthia Farquhar

New Zealand

Adesegun Fatusi

Nigeria

Shan-Wu Feng

China

Jennifer Fenwick

Australia

Alexandre Ferraro

Brazil

Cynthia Ferre

USA

Ginette Ferszt

USA

Catherine Field

Canada

Margaret Fisher

UK

Colleen Fitzgerald

USA

Niamh Fitzgerald

UK

Mirembe Florenc

Uganda

Elizabeth Ford

UK

Della Forster

Australia

Jean Christophe Fotso

Kenya

Karlyn Frank

South Africa

Abigail Fraser

UK

Eva Haukeland Fredriksen

Norway

Alexander Friedman

USA

May Friedman

Canada

Xavier Fritel

France

Roland Gaertner

Germany

Robin Gandley

USA

Joel Garbow

USA

Kefyalew Garie

Ethiopia

Vesna Garovic

USA

Deirdre Gartland

Australia

Caryl Gay

USA

Asheber Gaym

Ethiopia

Juliana Gebb

USA

Diederike Geelhoed

Mozambique

Ado Geidam

Nigeria

Stacie Geller

USA

Alan Gemmill

Australia

Alison Gernand

USA

Roslyn Carmel Giglia

Australia

Carmen Giurgescu

USA

Melissa Gladstone

UK

Lisa Gold

Australia

Robert Goldenberg

USA

Madalena Gomes Da Silva

Portugal

Luis Gomez

USA

Adrienne Gordon

Australia

Ingrid Granne

UK

David Griffith

Germany

Rosalie Grivell

Australia

Mechthild M. Gross

Germany

Tanja Groten

Germany

Susanne Grylka-Baeschlin

Germany

Ricardo Gurgel

Brazil

Annelie Gutke

Sweden

Mark Guy

UK

Rosalind Haddrill

UK

Batool Haider

USA

Thomas Hale

USA

Wendy Hall

Canada

Karin Hammarberg

Australia

Charlotte Hanlon

Ethiopia

Tharangrut Hanprasertpong

Thailand

Claudia Hanson

Sweden

Karen Hardee

USA

Mehmet Harma

Turkey

Joanne Harrold

UK

Merryl Harvey

UK

Haleema Hashmi

Pakistan

Sahar Hassan

Palestinian Territories

Toshiyuki Hata

Japan

Marie Hatem

Canada

Yvonne Hauck

Australia

Alys Havard

Australia

Donald Hayes

USA

Maureen Isabella Heaman

Canada

Alexander Heazell

UK

Paula Hedley

Denmark

Renee Heffron

USA

Jane Henderson

UK

Annemarie Hennessy

Australia

Amanda Henry

Australia

Bernardo Hernandez Prado

USA

Florian Herse

Germany

Nicola Heslehurst

UK

Leila Hessini

Morocco

Nicole Highet

Australia

Justus Hofmeyr

South Africa

Diane Holditch-Davis

USA

Michael Holick

USA

Caroline Hollins Martin

UK

Jennifer Hollowell

UK

Caroline Homer

Australia

Monjurul Hoque

South Africa

Muhammad Hoque

South Africa

Dell Horey

Australia

Shigeko Horiuchi

Japan

Lisa Howard-Grabman

USA

Daniel Hruschka

USA

Gang Hu

USA

Kun Huang

China

Carl Hubel

USA

Thomas Hulsey

USA

Tracy Humphrey

UK

Joke Aurelia Maria Hunfeld

Netherlands

Tai-Ho Hung

Taiwan

Lisa Hurt

UK

Julia Hussein

UK

Eileen Hutton

Canada

Lisa Hynes

Ireland

Adil Ibnouf

Saudi Arabia

Lawrence Ikamari

Kenya

Anastasia Iliadou

Sweden

Zubairu Iliyasu

Nigeria

Aamer Imdad

USA

Wan-Yim Ip

China

Nicola Jackson

Thailand

Vanitha Jagannath

Bahrain

B. Jager

Netherlands

Narayan Jana

India

Suze Jans

Netherlands

Susan Janson

USA

Patricia Janssen

Canada

Neau Jean-Philippe

France

Maria Jenmalm

Sweden

Janusz Jezewski

Poland

Mathew John

India

Kate Jolly

UK

Julie Jomeen

UK

Sue Jordan

UK

K Joseph

Canada

Anne Junker

Canada

Risto Kaaja

Finland

Brenda Kaaya

Namibia

Jerome Kabakyenga

Sweden

Marian Kacerovsky

Czech Republic

Udo Kaisers

Germany

Sumit Kane

Netherlands

Michael Katz

USA

Dan Kaye

Uganda

Gilles Kayem

France

Bengt Kayser

Switzerland

Miriam Keane

Australia

Sarah Keim

USA

Marc Jnc Keirse

Australia

Tony Kelly

UK

Jacqueline Kent

Australia

Heribert Kentenich

Germany

Remon Keriakos

UK

Inaam Khalaf

Jordan

Asma Khalil

UK

Mahmooda Khaliq

USA

Amina Khambalia

Australia

Iqbal Ansary Khan

Bangladesh

Ali S Khashan

Ireland

Selina Khatun

Bangladesh

Elaine Kidney

UK

Chong Jai Kim

South Korea

John Kinuthia

Kenya

Russell Kirby

USA

Ida Kirkegaard

Denmark

Michael Klein

Canada

Reija Klemetti

Finland

Vibeke Kildegaard Knudsen

Denmark

Thecla Kohi

Tanzania

Owonikoko Kola

Nigeria

Louis Kollee

Netherlands

Haruki Komatsu

Japan

Eugene Justine Kongnyuy

Cameroon

Justin Konje

UK

Klaas Koop

Netherlands

Jude Kornelsen

Canada

Steven Korzeniewski

USA

Andrew Kotaska

Canada

Vibeke Koushede

Denmark

Naoko Kozuki

USA

Alexander Krafft

Switzerland

Michael Kramer

USA

Hanne Kronborg

Denmark

Niranjana Krupa

Malaysia

Ganesh Kumar S

India

Juan Kusanovic

USA

Linda Kvist

Sweden

Barbara Kwast

Netherlands

Lars Ladfors

Sweden

Katariina Laine

Norway

Joanne Lally

UK

Joan Lalor

Ireland

Hugh Simon Hung San Lam

Hong Kong

Babbette Lamarca

USA

Geralyn Lambert-Messerlian

USA

Marcus D. Lancé

Netherlands

Helain Landy

USA

Isam Lataifeh

Jordan

Huynh-Nhu Le

USA

Nicky Leap

Australia

Alberto Leardini

Italy

James Lecheminant

USA

Meng-Chih Lee

Taiwan

Wesley Lee

USA

Ryan Lennon

USA

Felicia Lester

USA

Ky Leung

Hong Kong

Nan Li

USA

Adolfo Liao

Brazil

Edward Liechty

USA

Heiko Lier

Germany

Ching-Chiang Lin

Taiwan

Ge Lin

USA

Eva Lindberg

Sweden

Helena Lindgren

Sweden

Gary Lipscomb

USA

Robert Liston

Canada

James Litch

USA

Ching-Ming Liu

Taiwan

Cindy Liu

USA

Li Liu

USA

Sue Lo

Hong Kong

Terhi Lohela

Finland

Natalia Lokhmatkina

UK

Kinke Lommerse

Netherlands

Mary Longworth

UK

Mona Loutfy

Canada

Christine Loytved

Germany

Mirjam Lukasse

Norway

Pisake Lumbiganon

Thailand

Najaaraq Lund

Denmark

Ingela Lundgren

Sweden

Jennifer Lutomski

Ireland

Nanna Maaløe

Denmark

Alison Macfarlane

UK

Elizabeth Machado

Brazil

Karen Mackinnon

Canada

Everett Magann

USA

Moke Magoma

Tanzania

Abha Maheshwari

UK

Syed Mahmood

India

Emily Mailey

USA

Margaret C. Maimbolwa

Zambia

Franz Majoko

UK

Amitabha Majumdar

UK

Maria Yolanda Makuch

Brazil

Ahmad Makuwani

UK

Mats Malqvist

Sweden

Rosemary Mander

UK

Saknan Manotaya

Thailand

Karel Marsal

Sweden

Lena B Martensson

Sweden

Pamela Martey

Ghana

Gileard Masenga

Tanzania

Susan Mason

USA

Gabriele Materazzi

Italy

Matthews Mathai

Switzerland

Catriona Matheson

UK

Carol Mathews

USA

Thubelihle Mathole

South Africa

Zoe Matthews

UK

Robyn Maude

New Zealand

Maureen Mayhew

Canada

Alfred Mbah

USA

Anthony Mbah

Nigeria

Godfrey Michael Mbaruku

Tanzania

Columba K Mbekenga

Tanzania

Affette Mccaw-Binns

Jamaica

Candace Mcclure

USA

Elizabeth Mcclure

USA

Christine Mccourt

UK

Susan Mcdonald

Australia

Meredith Mcintyre

Australia

Elizabeth Mcpherson

USA

Tarek Meguid

Malawi

Jamila Mejdoubi

Netherlands

Sara Meltzer

Canada

Jan Mens

Netherlands

Fiona Mensah

UK

Maarit Mentula

Finland

Mario Merialdi

Switzerland

Marjo Metsäranta

Finland

Miriam Mgonja

Tanzania

Otilia Micle

Romania

Ornella Milanesi

Italy

Angela Miller

USA

Suellen Miller

USA

Renee Milligan

USA

Anne-Frederique Minsart

Belgium

Monika Mitra

USA

Yoshihiro Miyake

Japan

Kazuhiko Moji

Japan

Margareta Mollberg

Sweden

Rob Mooij

Netherlands

Tiffany Moore Simas

USA

Allisyn Moran

USA

Joan Morris

UK

Shaun Morris

USA

Michelle Mottola

Canada

Jean-Francois Mouillet

USA

Cheryl Moyer

USA

Ellen Mozurkewich

USA

Lilian Teddy Mselle

Tanzania

Sia Msuya

Tanzania

Projestine Muganyizi

Tanzania

Susmita Mukhopadhyay

India

Luke Mullany

USA

Zubia Mumtaz

Canada

Christiane Muth

Germany

Steve Mutiso

Kenya

Beatrice Mwagomba

Malawi

Ipyana Mwampagatwa

Tanzania

Bronwyn Myers

South Africa

Judith Myers

Australia

Gyula Richárd Nagy

Hungary

Jolly Nankunda

Uganda

Anwar Nassar

Lebanon

Natasha Nassar

Australia

Ellen Nelissen

Tanzania

Isabella Neri

Italy

Birgitte Bruun Nielsen

Denmark

Giovanni Nigro

Italy

Wendy Nilsen

Norway

Christina Nilsson

Sweden

Lena Nilsson-Wikmar

Sweden

Elie Nkwabong

Cameroon

Jane Norman

UK

Fred Nuwaha

Uganda

Uchenna Nwagha

Nigeria

Angelo Nyamtema

Tanzania

Pirkko Nykänen

Finland

Laura Oakley

UK

Colm Oboyle

Ireland

Rhoune Ochako

Kenya

Wendy Oddy

Australia

Ju Lee Oei

Australia

Tinuade Ogunlesi

Nigeria

Akihide Ohkuchi

Japan

Joseph Okeibunor

Congo

Michele Okun

USA

Morten Olsen

Denmark

Pia Olsson

Sweden

Bolajoko O. Olusanya

Nigeria

Abimbola Oluwatosin

Nigeria

Deirdre O'Malley

Ireland

Joseph Onakewhor

Nigeria

Stephen Onwere

Nigeria

Baafuor Opoku

Ghana

Peter O'Rourke

Australia

Rahmi Örs

Turkey

Carlos Ortega González

Mexico

Eugene Oteng-Ntim

UK

Ellis Owusu-Dabo

Ghana

Reena Oza-Frank

USA

Rebecca Painter

Netherlands

Kathryn Panaretto

Australia

Sarita Panday

Nepal

Vassiliki Papaevangelou

Greece

Mary Parpinelli

Brazil

Dharmintra Pasupathy

UK

Ulrich Pecks

Germany

Olavi Pelkonen

Finland

Andrea Barnabas Pembe

Tanzania

Juan Pablo Pena-Rosas

Switzerland

Debbie Penava

Canada

Suzanne Penfold

Tanzania

Gloria Pérez

Spain

Janette Perz

Australia

Antje Petersen

Germany

Manuela Pfinder

Germany

Victor Pop

Netherlands

Nick Powell

UK

Robert Powers

USA

Robert Powers

USA

Danielle Kaye Prime

Australia

Are Hugo Pripp

Norway

Peter Proff

Germany

Audrey Prost

UK

Marco Puccini

Italy

Xu Qian

China

Chunfang Qiu

USA

Xingda Qu

Singapore

Faisal Qureshi

USA

Mosiur Rahman

Bangladesh

Sajjad Rahman

UK

Parvin Rahnama

Iran

Sari Räisänen

Finland

Augustine Rajakumar

USA

Heribert Ramroth

Germany

Judith Rankin

UK

Chalapati Rao

Australia

Svein Rasmussen

Norway

Simmi Ratan

India

Jacques Ravel

USA

Joanna Raven

UK

Sundari Ravindran

India

Camille Raynes-Greenow

Australia

Maggie Redshaw

UK

Cavan Reilly

USA

Birgit Reime

Canada

Birgit Reime

Germany

Zilma Reis

Brazil

Mary Renfrew

UK

Jennifer Requejo

USA

Kathy Richards

USA

Yana Richens

UK

Russel Rickard

USA

Elisha Riggs

Australia

Marcus Rijken

Thailand

Marlies Rijnders

Netherlands

Christine Roberts

Australia

Sarah Roberts

USA

Yadira Roggeveen

Netherlands

Liliam Cristine Rolo Paiato

Brazil

Horace Roman

France

Kathryn Rose

Australia

Tessa Roseboom

Netherlands

Melissa Rosenstein

USA

Mary Ross-Davie

UK

Cristina Rossi

Italy

Beverly Rossman

USA

Ingrid Rowlands

Australia

Meera Roy

UK

Henrik Rueffert

Germany

Amelia Ruffatti

Italy

Stephen Rulisa

Rwanda

Kath Ryan

Australia

Riadh Sadik

Sweden

Sarah Saleem

Pakistan

Sudha Salhan

India

Raed Salim

Israel

Kirsten Salmeen

USA

Anne Salonen

Finland

Jane Sandall

UK

Harshadkumar Sanghvi

USA

Ayesha Sania

USA

Despina Sapountzi-Krepia

Greece

Narendra Nath Sarkar

India

Malabika Sarker

Bangladesh

Stephen Sawiak

UK

Barbara Scavone

USA

Timme Schaap

Netherlands

Ashley Schempf

USA

Lone Schmidt

Denmark

Virginia Schmied

Australia

Mavis Schorn

USA

Jane Scott

Australia

Neil Scott

UK

Lisa Segre

USA

Hen Sela

USA

Katherine Semrau

USA

Valerie Seror

France

Laura Serrant-Green

UK

Suzanne Serruya

Uruguay

Kashif Shafique

UK

Richard Shaw

UK

Lumaan Sheikh

Pakistan

Julia Shelley

Australia

Zoe Sheppard

UK

Mary Sheridan

UK

Daudi Simba

Tanzania

Kavita Singh

USA

Yuri Sinzato

Brazil

Janna Skagerström

Sweden

Alkistis Skalkidou

Sweden

Lillian Skibsted

Dominican Republic

Nancy L Sloan

USA

Nancy L Sloan

USA

Rhonda Small

Australia

Jeffrey Michael Smith

USA

Valerie Smith

Ireland

Jonathan Snowden

USA

Malin Soederberg

Sweden

Andrea Solnes Miltenburg

Netherlands

Werner Soors

Belgium

David Soper

USA

Eleazar Soto

USA

Joao Paulo Souza

Brazil

Sydney Spangler

USA

Kevin Spencer

UK

Chandrashekhar Sreeramareddy

Nepal

Tomasina Stacey

UK

Cynthia Stanton

USA

Anke Steckelberg

Germany

Eric Steegers

Netherlands

Regine Steegers-Theunissen

Netherlands

Vedran Stefanovic

Finland

Elizabeth Stenhouse

UK

Olof Stephansson

Sweden

Joanna Stewart

New Zealand

Kathrin Stoll

Canada

Gretchen Stuart

USA

Kent Stuber

Canada

Peter Stutchfield

UK

Sa'Adatu Sule

Nigeria

Reijo Sund

Finland

Johanne Sundby

Norway

Fernanda G C Surita

Brazil

Elisabeth Svensson

Sweden

Linda Sweet

Australia

Henock Taddese

UK

Peng Chiong Tan

Malaysia

Tara Tancred

UK

David Tappin

UK

Lesley Tarasoff

Canada

Belaynew Wasie Taye

Ethiopia

Kate Teela

USA

Cristina Teixeira

Portugal

Nguyen Thi Thuy Hanh

Vietnam

Liu Lin Thio

USA

Gill Thomson

UK

Stina Thorstensson

Sweden

Severine Thys

Belgium

Christiana Titaley

Indonesia

Catherine Todd

USA

Fahmida Tofail

Bangladesh

Paloma Toledo

USA

Stephen Tong

Australia

Van Tong

USA

Gabriele Tonni

Italy

Maria Regina Torloni

Brazil

Clare Tower

UK

Vandana Tripathi

USA

Athanasios Tselebis

Greece

Panagiotis Tsikouras

Greece

Jamilu Tukur

Nigeria

Ozge Tuncalp

USA

Volkan Turan

Turkey

Odi' Ugochukwu Joannes Umeora

Nigeria

Regine Unkels

UK

Jukka Uotila

Finland

Ihab Usta

Lebanon

Michele Usuelli

Italy

Bettina Utz

UK

Yavuz Uyar

Turkey

Edi Vaisbuch

USA

Edimarlei Gonsales Valerio

Brazil

Jodie Valpied

Australia

Philippe Van De Perre

France

Thomas Van Den Akker

Netherlands

Olivier Van Der Heijden

Netherlands

Karin Van Der Tuuk

Netherlands

Karen Van Der Veken

Belgium

Jeroen Van Dillen

Netherlands

Paul Van Kesteren

Netherlands

Edwin Van Teijlingen

UK

Christiaan Van Woerden

Netherlands

Jacob Van Wouwe

Netherlands

Jeroen Vanderhoeven

USA

Paula Vargas Innocenti

Chile

Rajesh Varma

UK

Katri Vehvilainen-Julkunen

Finland

Maria P. Vélez

Canada

Douwe Verkuyl

Netherlands

Stefan Verlohren

Germany

Tienke Vermeiden

Ethiopia

Maria Cristina Verrocchio

Italy

Georgia Verropoulou

Greece

Jenny Vieveen

Netherlands

Simone Vigod

Canada

Sarah Fredsted Villadsen

Denmark

Naomi Vink

Netherlands

Jolande Vis

Netherlands

Nina Vollestad

Norway

Frauke Von Versen-Höynck

Germany

Fred Vork

Netherlands

Gail Wade

USA

Yohannes Dibaba Wado

Ethiopia

Carol Wagner

USA

Hayfaa Wahabi

Sudan

Peter Waiswa

Uganda

L. Lewis Wall

USA

Denis Walsh

UK

Julia Walsh

USA

Fuzhou Wang

China

Yuping Wang

USA

Charlotte Warren

Kenya

Lucie Warren

UK

Lyndsey Watson

Australia

Gary Webb

USA

G Webster

Canada

Andrew Weeks

UK

Carolyn Weiniger

Israel

Adi Weintraub

Israel

Tracey Weissgerber

USA

Eliana Wendland

Brazil

Nedra Whitehead

USA

Heather Whitford

UK

Therese Wiegers

Netherlands

Carol Wilkins

UK

Shelley Wilkinson

Australia

Andrew Willan

Canada

Jonathan Williams

UK

Melissa Wilson

USA

Deanne Wilson-Costello

USA

Tom Witteveen

Netherlands

Stephen Wood

Canada

Hannah Woolhouse

Australia

Andrzej Wykretowicz

Poland

Bin Xie

Canada

Ibrahim Yakasai

Nigeria

Mohammad Yakoob

USA

Junta Yanai

Japan

Huixia Yang

China

Faith Yego

Kenya

Edwina Yeung

USA

Takashi Yorifuji

Japan

David Young

Canada

Yilmaz Yozgat

Turkey

Eivind Ystrom

Norway

Hong Wa Yung

UK

Agnieszka Zawiejska

Poland

Lingxia Zeng

China

Lei Zhang

USA

Jing Zheng

USA

Birute Zilaitiene

Lithuania

Nadeem Zuberi

Pakistan

Dejan Zurovac

Kenya

Prisca Ac Zwanikken

Netherlands

## Pre-publication history

The pre-publication history for this paper can be accessed here:

http://www.biomedcentral.com/1471-2393/14/18/prepub

